# Uric Acid from Day to Day

**Published:** 1917-11

**Authors:** John C. Warbrick

**Affiliations:** Chicago


### Uric Acid From Day to Day.

By JOHN C. WARBRICK, M. D., Chicago.

It has often been stated that eating meat is injurious, that it is poisonous and that it is the chief agent in causing uric acid to form in the body.

Judging from these statements one would almost be led to believe at first that uric acid is a real factor in causing some of the numerous diseases of the body.

Dr. Haig of London, Eng., supposed to be the greatest authority in the world on the subject of uric acid, has for many years along with a few others denounced meat as being harmful and as the real cause in the production of uric acid in the body.

When these statements are made by an authority recognized the whole world over such as Dr. Haig, and which no doubt many medical men have believed for years to be true, it would seem then possibly to be of little or no avail for anyone to state anything different on the subject.

Dr. Haig it seems has examined thousands of specimens of urine to prove that his theory is correct, which may be the case according to the way he looks at the matter, and I suppose it would hardly be of any use in trying to convince him, there is another side to the question, and that sufficient evidence can be produced to prove his statements are not in accordance with the facts.

It may be stated, and it has been proved many times, that it is not necessary to have uric acid in the system, simply because one is accustomed to eating meat.

From my long experience in examining specimens of urine I have found that the presence of uric acid is not nearly so common as might be thought. Anyway if a little uric acid is occasionally found in the urine it can hardly be considered of much consequence and does not tend to produce marked symptoms of any kind judging from close observation.

It is somewhat of a puzzle for me to understand just how Dr. Haig came to the conclusion he has regarding uric acid, and then to have them accepted generally as correct, while all other theories it would seem are of no account.

The mere statement that eating meat causes uric acid to form in the body is not based upon facts, nor is it consistent with scientific reasoning of any breadth of thought, so it must therefore be considered somewhat erroneous as can and has been proved more than once.

Under certain conditions it may be said, the eating of meat may cause some uric acid to form in the body, and under certain conditions it may not do so. Haig's theory, it seems to me, is rather one sided, and not in accordance with the real facts of the matter, for he has looked into the subject in one way only.

After some careful investigations along this line, I have came to the conclusion that meat can be eaten regularly, and more so under certain conditions, without having any uric acid whatever form in the body.

In order to find out just what amount of uric acid, if any at all would be found in specimens of urine taken from day to day for a time and examined carefully after a meal of meat had been eaten, I made the following experiment:

I put a man about forty years old, of full eating habit and a consumer of more or less proteid material all the time, on a diet for several days.

He was to take meat at his meals once or twice a day, as much as he wished, drink water in between his meals and keep his bowels well open.

A short time after his food had been taken he was to collect a sample of his urine for examination.

If uric acid occurred in the urine of any one, it would be most likely to be found in this subject, a married man, on his feet a great deal, inside mostly and not getting much exercise.

He followed out the instructions given him and collected a sample of his urine for about six days regularly. These six specimens of urine were considered sufficient to prove whether the eating of meat would cause any uric acid to form in the body or not.

The urine was examined in detail each time, but mostly to find the amount of chlorides, phosphates and sulphates present and to make the sediment carefully as to the formation of crystals.

Out of the six careful urinary tests made, not a single crystal of uric acid could be detected by microscopic examination, and it was hardly to be expected that any traces would be found under the circumstances.

The color of the urine was yell. red in every instance but one, while the sp. gr. in each report made was low and no higher than 1020, no lower than 1018. Under this method the chlorides never reached a high figure at any of the examinations made as might only be expected. The highest point reached was at 20—310 in one instance, just a little beyond the normal daily maximum amount.

In five instances the amount of chlorides was very low and especially so in one case.

Upon five occasions the amount of chlorides was found to be below the normal daily maximum amount, and three tmes just equal to the minimum daily figure, while once they were much beneath the normal daily minimum amount at the figure 5—77.5 which one would hardly expect.

In order to find out further whether or not any uric acid would be noted in the urine from eating proteid food, such as meat especially, I made some routine experiments upon myself for a few days.

I took a good meal of meat regularly once a day and afterwards collected a sample of the urine for examination. This was kept up for about eight days, a specimen of urine being collected after each meal.

Other articles of food were taken as well at each meal, while water was drunk in between them.

Altogether eight specimens of urine were carefully examined and the results were tabulated as follows:

The color in every case was yellowish red. The sp. gr. was high during the course of diet, reaching upon two occasions the unusually high mark at 1042, while the average was about 1030, and only once did it get below this figure at 1025.

The amount of chlorides never reached a high figure at anytime during the examinations, ranging from the low figure 8—124 below the minimum daily amount upon two occasions to the fairly high mark at 25—387.5 found only once. About five times the amount of chlorides was found to be low and beneath the normal daily maximum amount. The amount of phosphates was about normal in three instances at the usual maximum figure, while only once did they exceed the normal maximum index and oniy once registered below it.

The amount of sulphates did not reach a very high mark at any of the examinations. In five instances they were below the normal minimum daily index at the figure one, while in only a single case did they reach the maximum daily amount. Where much proteid food is taken regularly, such as meat and eggs, if all of the tissues of the body are functioning normally and properly, an increase in the amount of chlorides may be looked for almost as the rule, and the same in case of the sulphates which has often been noted.

The amount of sulphates of course is more or less dependent upon the intake of proteid food as to whether it is at a low or a high figure.

In the present cases however, where proteid food has been taken daily, it has been found that both the amount of chlorides and sulphates are low, so that under these circumstances the presence of uric acid would hardly be looked for in the urine.

In these reports the amount of sulphates especially has to be noted, for in some instances they reached a very high point that is seldom noted, ranging all the way from the low figure 3—46.5 to the unusual index at 50—775.

In five instances the amount of sulphates was high and much beyond the normal daily maximum figure, while in three cases only, the point was just equal to the maximum daily register.

The next highest amount of sulphates was at the mark 30—465, which occurred twice, a point far beyond the normal index.

On three occasions the amount of sulphates was found to exceed a good deal the highest point reached by the chlorides at any of the examinations.

The register of the phosphates was high at all of the tests but one, exceeding by a good deal the normal daily maximum amount. With both the phosphates and sulphates at such a high mark in each case, it would almost seem as if uric acid would be present in the urine, still this was not the case, even if there was tissue sub-oxidation, causing it it will be seen, the sp. gr. to be unusually high a number of times.

1. Yell. red. Sp. gr. 1020; Cls.—10:155; P.—5:77.5; S.—1:15.5; Uric acid—0.

2. Yell. red. Sp. gr. 1020; Cls.—10:155; P.—5:77.5; S.—½:8.

3. Yell. red. Sp. gr. 1018; Cls.—10:155; P.—3:46.5; S.—1:15.5.

4. Yell. red. Sp. gr. 1018; Cls.—20:310; P.— 10:155; S.—1:15.5.

5. Yell. red. Sp. gr. 1020; Cls.—5:77.5; P.—0:0; S.—3:46.5.

6. Light yell. Sp. gr. 1018; Cls.—15:232.5; P.—5:77.5; S.—1:15.5.

After eating meat:

1. Yell. red. Sp. gr. 1025; Cls. 10:155; P.—2:31; S.—30:465; Uric acid—0.

2. Yell. red. Sp. gr. 1042. Cls.—10:155; P.—20:310; S.—15:232; Uric acid—0.

3. Yell. red. Sp. gr. 1030; Cls.—20:310; P.—10:155; S.—3:46.5; Uric acid—0.

4. Yell. red. Sp. gr. 10:30; Cls. 20:310; P.—15:232; S.—3:46.5; Uric acid—0.

5. Yell. red. Sp. gr. 1032; Cls.—25:387.5; P.—20:310; S.—3:46.5; Uric acid—0.

6. Yell. red. Sp. gr. 1035; Cls.—10:155; P.—15:232.5; S.—20:310; Uric acid—0.

7. Yell. red. Sp. gr. 1034; Cls.—8:124; P.—15:232.5; S.—30:465.

8. Yell. red. Sp. gr. 1042; Cls.—8:124; P.—8:124; S.—50:775.

---

**Spirochaetes.** Hideo Noguchi of N. Y. in the Jour. of Lab. & Clin. Med., Mch., 1917, reviews at length, the biologic classification and relation to various diseases in man and animals of the surprisingly large list of species and discusses the viability, susceptibility to antiseptics and other factors, especially with regard to treponema pallidum. With special cultural precautions, including attention to temperature, provision of pabulum and recultivation, T. pallidum may survive a year or more but, without special precautions, even in its original site in post mortem material, it usually dies in 1-3 days. It is killed by temperatures of 50-55° C. within 20 minutes, or according to Akatsu, it may survive an hour at 60° and 10 minutes at 55° but is killed at 55° within 15 minutes and at 60° and upward, within 5 minutes. Tables show that it is killed by a large variety of antiseptics and even drugs not usually so considered, in dilutions much weaker than those commonly used. Among these are the bile salts and sodium oleinicum. (Other reports state that ordinary soap quickly kills and that low temperatures at least markedly reduce the virulence). The general conclusion seems to be justified that syphilis is a disease in which fomites in the ordinary sense, play a small part and that it is not liable to transmission by faults of sanitation; in other words, that discharges are not apt to produce the disease unless by very direct transference. These facts are in agreement with purely empiric observations as to methods of infection. While a considerable proportion of cases are "insontium," analysis shows that the term must be interpreted in a quite strict moral sense. The majority of cases of syphilis insontium are due to the recognized common etiologic factor which, of course, is equally dangerous with or without a marriage certificate, if the other party has the disease. The majority of the remaining cases of syphilis insontium are due to close personal contact, including indeed caresses of a strictly sexual nature, other than coitus. In a good many cases the term "insontium" is a misnomer and merely recognizes certain arbitrary distinctions of personal contact under sexual excitement. The plain fact of the matter is that strictly scientific research fully justifies empiric scepticism of the probability of contracting syphilis by ordinary accidents.

